# Supplementary material for: The interactome of intact mitochondria by cross-linking mass spectrometry provides evidence for coexisting respiratory supercomplexes
Source: Mol Cell Proteomics. 2017 Dec 8;17(2):216–32. doi: 10.1074/mcp.RA117.000470 (PMC5795388; doi:10.1074/mcp.RA117.000470)
Supplement: Supplemental Data [file supp_RA117.000470_133922_0_supp_23978_fzffwf.zip › spectra_annotation/mito_spectra_annotation/488-1-4-1-8-1.pdf]

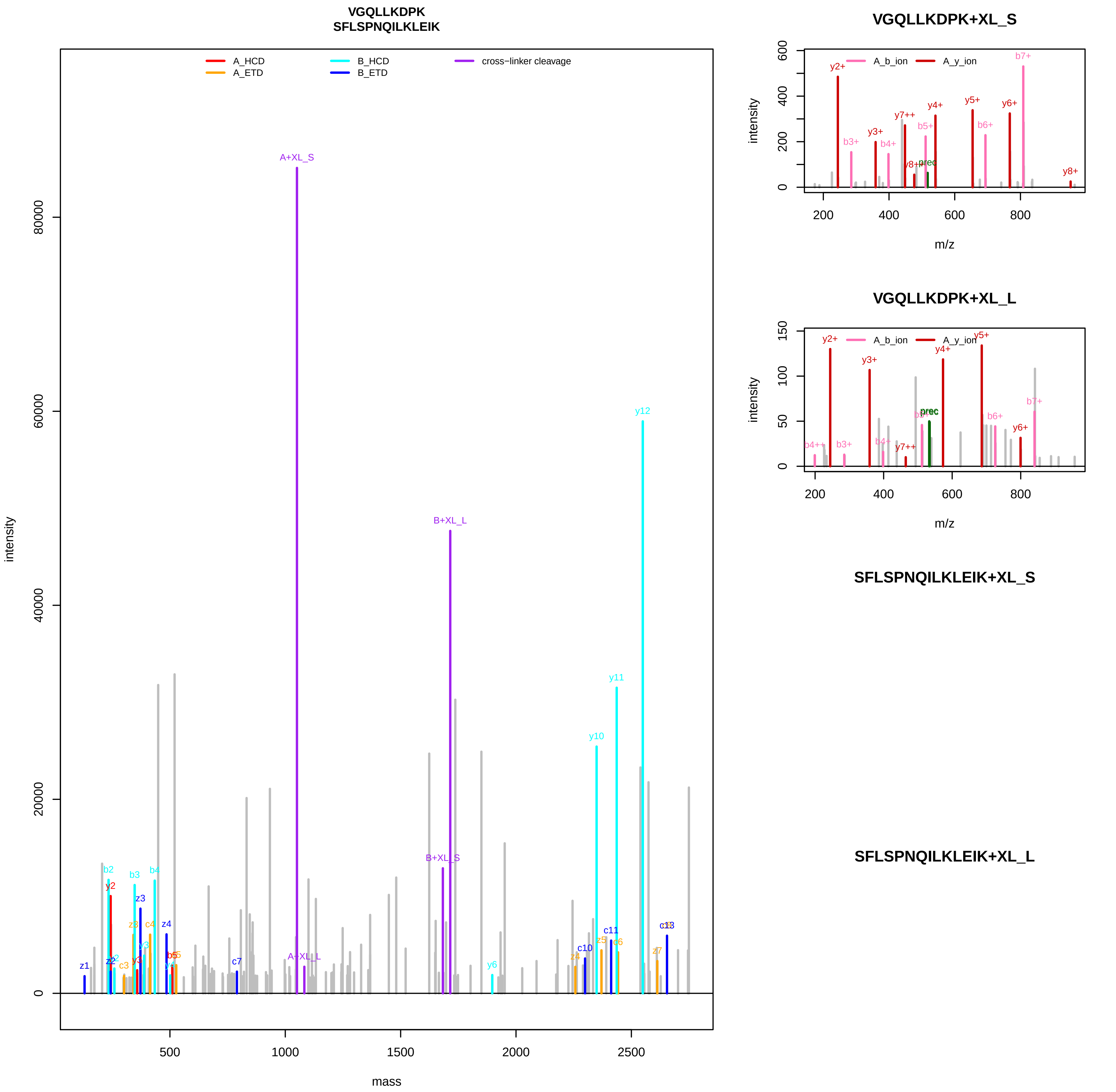
VGQLLKDPK
SFLSPNQILKLEIK
A_HCD
A_ETD
B_HCD
B_ETD
cross−linker cleavage
A+XL_S
B+XL_L
B+XL_S
A+XL_L
y12
y11
y10
y6
b2
b3
b4
y2
y3
z1
z2
z3
z4
c3
c4
c7
c10
c11
c13
z5
z4
c6
z7
b5
intensity
mass
0
20000
40000
60000
80000
500
1000
1500
2000
2500
VGQLLKDPK+XL_S
A_b_ion
A_y_ion
y2+
y3+
y4+
y5+
y6+
y7++
y8+
b3+
b4+
b5+
b6+
b7+
prec
intensity
m/z
0
200
400
600
800
VGQLLKDPK+XL_L
b4++
b3+
b4+
b6+
b7+
y2+
y3+
y4+
y5+
y6+
y7++
prec
0
50
100
150
200
400
600
800
SFLSPNQILKLEIK+XL_S
SFLSPNQILKLEIK+XL_L
